# Global dynamics for a class of discrete SEIRS epidemic models with general nonlinear incidence

**DOI:** 10.1186/s13662-016-0846-y

**Published:** 2016-05-06

**Authors:** Xiaolin Fan, Lei Wang, Zhidong Teng

**Affiliations:** 1 College of Mathematics and System Sciences, Xinjiang University, Urumqi, 830046 People’s Republic of China; 2 Department of Basic Education, Xinjiang Institute of Engineering, Urumqi, 830091 People’s Republic of China; 3 Department of Medical Engineering and Technology, Xinjiang Medical University, Urumqi, 830054 People’s Republic of China

**Keywords:** 37M99, 39A11, 92D30, discrete SEIRS epidemic model, nonlinear incidence, basic reproduction number, global attractivity, permanence

## Abstract

In this paper, a class of discrete SEIRS epidemic models with general nonlinear incidence is investigated. Particularly, a discrete SEIRS epidemic model with standard incidence is also considered. The positivity and boundedness of solutions with positive initial conditions are obtained. It is shown that if the basic reproduction number $\mathcal{R}_{0}\leq1$, then disease-free equilibrium is globally attractive, and if $\mathcal{R}_{0}> 1$, then the disease is permanent. When the model degenerates into SEIR model, it is proved that if $\mathcal{R}_{0}> 1$, then the model has a unique endemic equilibrium, which is globally attractive. Furthermore, the numerical examples verify an important open problem that when $\mathcal{R}_{0}>1$, the endemic equilibrium of general SEIRS models is also globally attractive.

## Introduction

As is well known, many infectious diseases possess a latent period, such as Hepatitis, HIV, SARS, Ebola, MERS, *etc.* When a susceptible individual is infected at the beginning, the disease incubates inside the susceptible for a period of time, then the susceptible becomes an exposed individual before becoming infectious. For such infectious diseases, the resulting model is SEIR (susceptible *S*, exposed *E*, infectious *I*, removed *R*) epidemic type. The study on SEIR-type epidemic dynamical models is a very important subject in the mathematical theory of epidemiology, and in the last two decades there have been a number of researches on modeling, theoretical analysis, and applications. Continuous SEIR-type epidemic models described by the differential equations have been widely studied. Many important and interesting results can be found in [1–9] and the references therein.

As we all know, it is very difficult to accurately solve a nonlinear differential equation with a given initial condition. Therefore, for many practical requirements, such as numerical calculation, it is often necessary to discretize a continuous model to obtain the corresponding discrete model. At the present time, there are various discretization methods to discretize a continuous model, including the standard methods, such as Euler method, Runge-Kutta method, and some other standard finite difference schemes, and the nonstandard finite difference (NSFD) scheme, which is originally developed by Mickens [10–12].

In recent years, discrete epidemic models have been widely studied. The basic and important research subjects for these models are the computing of the thresholds values and basic reproduction numbers, the local and global stability of disease-free equilibrium and the endemic equilibrium, the persistence, permanence, and extinction of the disease, and bifurcations and chaos phenomena of the models when some parameters of the models vary, and so on. Many important and interesting results can be found in [13–36] and the references therein. Particularly, we see that in [14, 17, 18, 20–22, 27, 36] discrete SI-type epidemic models are investigated, and in [19, 24, 25, 31, 32, 35] discrete SIR-type epidemic models are discussed.

However, we see that up to now there have been fewer research works on discrete SEI- and SEIR-type epidemic models, where the disease has a latent period. Cao and Zhou [16] formulated and studied a discrete age-structured SEIT epidemic model, and as an application, discussed the tuberculosis transmission in China. In [26], the authors applied Micken’s discretization method to obtain a discrete SEIR epidemic model. The positivity of solutions and the existence and stability of equilibrium are discussed. The design of a state observer for the model is tackled. Some sufficient conditions to ensure the asymptotic stability of the observer are provided in terms of a matrix inequality. In [28], the authors studied a discrete plant virus disease model with roguing and replanting, which is derived from the continuous case by using the backward Euler method. The basic reproduction number $R_{0}$ is obtained. It is showed that the disease-free equilibrium is globally attractive if $R_{0}\leq1$, and otherwise, the disease is permanent if $R_{0}>1$. In [29, 30], the authors proposed a class of discrete SEIS epidemic models with bilinear incidence, which is established from the corresponding continuous SEIS epidemic model by applying the well-known backward difference scheme. The positivity of solutions and the permanence of the model are established. Furthermore, using the Lyapunov function method, the authors proved that if the basic reproduction number $R_{0}\leq1$, then the disease-free equilibrium is globally asymptotically stable, and if $R_{0}>1$, then the endemic equilibrium exists and is globally asymptotically stable.

Consider the following continuous SEIRS epidemic model with general nonlinear incidence:

1 $$ \left \{ \textstyle\begin{array}{@{}l} \frac{dS}{dt}= \Lambda-f(S,E,I,R)-\mu_{1}S+\sigma R,\\ \frac{dE}{dt}= f(S,E,I,R)-(\mu_{2}+\delta)E,\\ \frac{dI}{dt}= \delta E-(\mu_{3}+\gamma)I,\\ \frac{dR}{dt}= \gamma I-(\mu_{4}+\sigma)R. \end{array}\displaystyle \right . $$

Some particular cases for this model have been investigated in [3, 4, 26], where the basic reproduction number is calculated, and the dynamical properties, such as the local and global stability of disease-free equilibrium and endemic equilibrium and the extinction and persistence of the disease are established. Motivated by this work, in this paper, we propose the following discrete SEIRS epidemic model with general nonlinear incidence established by using the backward difference scheme to discretize model (1):

2 $$ \left \{ \textstyle\begin{array}{@{}l} S(n+1)-S(n)= \Lambda-f(X(n+1))-\mu_{1} S(n+1)+\sigma R(n+1),\\ E(n+1)-E(n)= f(X(n+1))-(\mu_{2}+\delta)E(n+1),\\ I(n+1)-I(n)= \delta E(n+1)-(\mu_{3}+\gamma)I(n+1),\\ R(n+1)-R(n)= \gamma I(n+1)-(\mu_{4}+\sigma)R(n+1), \end{array}\displaystyle \right . $$

where $X(n)=(S(n),E(n),I(n),R(n))$.

Our purpose in this paper is to investigate the dynamical behaviors of model (2). The basic reproduction number $\mathcal{R}_{0}$ is defined. We will prove by using the linearization method and Lyapunov function that if $\mathcal{R}_{0}\leq1$, then disease-free equilibrium is globally attractive, and as a result, the disease is also extinct, and by using the theory of persistence for dynamical systems that if $\mathcal {R}_{0}>1$, then the disease is permanent. Furthermore, when model (2) degenerates into the particular case $f(S,E,I,R)=f(S,E,I)$ and $\sigma=0$, by constructing the suitable discrete type Lyapunov function we also will prove that if $\mathcal {R}_{0}>1$, then model (2) has a unique endemic equilibrium, which is globally attractive.

The organization of this paper is as follows. In Section 2, the model description and some basic properties are given. Section 3 deals with the global attractivity of disease-free equilibrium of model (2). In Section 4, the criterion on the permanence of the disease for model (2) is stated and proved. In Section 5, the criterion on the global attractivity of the endemic equilibrium for model (2) in the particular case $f(S,E,I,R)=f(S,E,I)$ and $\sigma=0$ is stated and proved. Furthermore, in Section 6, some numerical examples are provided to illustrate the validity of main results obtained in this paper and verify the interesting open problem given in Remark 5.1. Lastly, a discussion is given in Section 7.

## Basic properties

In model (2), $S(n)$, $E(n)$, $I(n)$, and $R(n)$ denote the numbers of susceptible, exposed, infectious, and recovered classes at *n*th generation, respectively, Λ is the recruitment rate of the susceptible, $\mu_{i}$ ($i=1,2,3,4$) are the death rates of susceptible, exposed, infectious, and recovered individuals, respectively. Particularly, $\mu_{3}$ includes the natural death rate and the disease-related death rate of the infectious class. *δ* is the translation rate from exposed to infectious, *γ* is the recovery rate of the infectious individuals, and *σ* is the rate of losing immunity of the recovered; $\sigma>0$ indicates that the recovered individuals possess the provisional immunity, and $\sigma=0$ predicates that the recovered individuals acquire permanent immunity. The incidence rate of the infectious is described by a nonlinear function $f(S,E,I,R)$.

In this paper, we always assume that the parameters Λ, $\mu_{i}$ ($i=1,2,3,4$), *δ*, and *γ* are positive constants, *σ* is a nonnegative constant, and $\mu_{1}\leq\min\{\mu_{2},\mu_{3},\mu_{4}\}$. We set

$$\Omega=\bigl\{ (S,E,I,R,): S\geq0, E\geq0, I\geq0, R\geq0, S+E+I+R>0\bigr\} . $$

For a nonlinear incidence $f(S,I)$, we introduce the following assumption.

(H): $f(S,E,I,R)$ is continuously differentiable with respect to $(S,E,I,R)\in\Omega$, $f(S,E,I,R)$ is increasing with respect to $S\geq0$ and decreasing with respect to $E\geq0$ and $R\geq0$, and $\frac{f(S,E,I,R)}{I}$ is nonincreasing with respect to $I>0$. Furthermore, $f(0,E,I,R)=f(S,E,0,R)\equiv0$ and $\frac{\partial f(S_{0},0,0,0)}{\partial I}>0$, where $S_{0}=\frac{\Lambda }{\mu_{1}}$.

### Remark 2.1

When $f(S,E,I,R)=\beta\frac{S^{q}I}{(1+\omega S)(1+\alpha I^{p})}$ or $f(S,E,I,R)=\beta\frac{SI}{N}$, where $N=S+E+I+R$, and $\beta>0$, $\omega\geq0$, $\alpha\geq0$, $q\geq1$, and $p\geq0$ are constants, (H) naturally holds. Furthermore, when $f(S,E,I,R)=\beta h(S)g(I)$, (H) degenerates into the following form:

(H^∗^): $h(S)$ and $g(I)$ are continuously differentiable with respect to $S\geq0$ and $I\geq0$, respectively, $h(S)$ is increasing for $S\geq0$, and $\frac{g(I)}{I}$ is nonincreasing for $I>0$. Furthermore, $h(0)=g(0)=0$ and $g'(0)>0$.

The initial condition for model (2) is given by

3 $$ S(0)>0, \qquad E(0)>0,\qquad I(0)>0, \qquad R(0)\geq0. $$

We have the following result on the positivity and ultimate boundedness of solutions.

### Theorem 2.1

*Model* (2) *has a unique positive solution* $(S(n),E(n),I(n),R(n))$ *for all* $n\geq0$ *with initial condition* (3), *and this solution is ultimately bounded*.

### Proof

We can prove this theorem by using an argument similar to that introduced in [33], Theorem 2.2. In fact, we only need to prove by induction that, for any integer $n\geq0$, if $(S(n),E(n),I(n),R(n))$ exists and $S(n)>0$, $E(n)>0$, $I(n)>0$, and $R(n)\geq0$, then $(S(n+1),E(n+1),I(n+1),R(n+1))$ also exists, and $S(n+1)>0$, $E(n+1)>0$, $I(n+1)>0$, and $R(n+1)>0$.

From model (2) by calculating we can obtain

4 $$ S(n+1)=a-bE(n+1), \qquad I(n+1)=\frac{1}{1+\mu_{3}+\gamma}\bigl[I(n)+\delta E(n+1) \bigr], $$

and

5 $$ R(n+1)=\frac{1}{1+\mu_{4}+\sigma}\biggl[R(n)+\frac{\gamma}{1+\mu_{3}+\gamma }\bigl(I(n)+\delta E(n+1) \bigr)\biggr], $$

where

$$\begin{aligned} a={}& \frac{1}{1+\mu_{1}}\bigl(N(n)+\Lambda\bigr) - \biggl[\frac{\mu_{3}-\mu_{1}}{(1+\mu_{1})(1+\mu_{3}+\gamma)}+ \frac{1}{1+\mu _{3}+\gamma} \\ &{} +\frac{\gamma}{(1+\mu_{3}+\gamma)(1+\mu_{4}+\sigma)} +\frac{(\mu_{4}-\mu_{1})\gamma}{(1+\mu_{1})(1+\mu_{3}+\gamma)(1+\mu_{4}+\sigma )} \biggr]I(n) \\ &{} - \biggl[\frac{\mu_{4}-\mu_{1}}{(1+\mu_{1})(1+\mu_{4}+\sigma)} +\frac{1}{1+\mu_{4}+\sigma} \biggr]R(n), \\ b={}& \frac{1}{1+\mu_{1}} \biggl[\mu_{2}-\mu_{1} + \frac{(\mu_{3}-\mu_{1})\delta}{1+\mu_{3}+\gamma} +\frac{(\mu_{4}-\mu_{1})\gamma\sigma}{(1+\mu_{3}+\gamma)(1+\mu_{4}+\sigma)} \biggr] \\ &{} +1+\frac{\delta}{1+\mu_{3}+\gamma} +\frac{\gamma\delta}{(1+\mu_{3}+\gamma)(1+\mu_{4}+\sigma)}, \end{aligned}$$

and $N(n)=S(n)+E(n)+I(n)+R(n)$. Since $\mu_{1}\leq\min\{\mu_{2},\mu_{3},\mu _{4}\}$, we obtain $b>0$ and $a>\frac{1}{1+\mu_{1}}[S(n)+E(n)+\Lambda]>0$.

Let $y=E(n+1)$. By the second equation of model (2) and by (4) and (5), *y* satisfies the equation

$$\Phi(y)\triangleq y-\frac{1}{1+\mu_{2}+\delta} \bigl[E(n)+f\bigl(a-by,y,u(y),v(y)\bigr) \bigr]=0, $$

where

$$u(y)=\frac{1}{1+\mu_{3}+\gamma}\bigl(I(n)+\delta y\bigr) $$

and

$$v(y)=\frac{1}{1+\mu_{4}+\sigma}\biggl[R(n)+\frac{\gamma}{1+\mu_{3}+\gamma }\bigl(I(n)+\delta y\bigr) \biggr]. $$

Let $y_{0}=\frac{a}{b}$. Since

$$\Phi(y)=y-\frac{1}{1+\mu_{2}+\delta} \biggl[E(n)+\frac {f(a-by,y,u(y),v(y))}{u(y)}u(y) \biggr], $$

from (H) we obtain that $\Phi(y)$ is increasing with respect to $y\in(0,y_{0})$. Then, we obtain

$$\Phi(0)=-\frac{1}{1+\mu_{2}+\delta} \bigl[E(n)+f\bigl(a,0,u(0),v(0)\bigr) \bigr]< 0 $$

and

$$\Phi(y_{0})=y_{0}-\frac{1}{1+\mu_{2}+\delta}E(n)>0. $$

Therefore, $\Phi(y)=0$ has a unique positive solution $\bar{y}\in(0,y_{0})$. This shows that $E(n+1)$ exists and $E(n+1)=\bar{y}>0$.

By (4), when $E(n+1)>0$ exists, then $I(n+1)$ also exists, and $I(n+1)>0$. By the fourth equation of model (2) we further have that $R(n+1)$ exists and $R(n+1)>0$.

Let $x=S(n+1)$. By the first equation of model (2) it follows that

$$\Psi(x)\triangleq(1+\mu_{1})x+f\bigl(x,E(n+1),I(n+1),R(n+1)\bigr)- \sigma R(n+1)-S(n)-\Lambda=0. $$

By (H), when $E(n+1)>0$ exists, then $\Phi(x)$ is increasing for $x\geq0$. Since $\Psi(0)=-\sigma R(n+1)-S(n)-\Lambda<0$ and $\lim_{x\to\infty}\Psi(x)=\infty$, we obtain that $\Psi(x)=0$ has a unique positive solution *x̄*. Therefore, $S(n+1)$ exists, and $S(n+1)=\bar{x}>0$.

From the previous discussions we finally obtain that $(S(n+1),E(n+1),I(n+1), R(n+1))$ exists and is positive. Therefore, solution $(S(n),E(n),I(n),R(n))$ uniquely exists and is positive for all $n>0$.

From model (2) we have

$$N(n+1)\leq\frac{1}{1+\mu_{1}} \bigl[N(n)+\Lambda \bigr]. $$

When $N(0)\leq S_{0}$, where $S_{0}=\frac{\Lambda}{\mu_{1}}$, we have $N(n)\leq S_{0}$ for all $n>0$. In a general way, for any $N(0)>0$ we can obtain $\limsup_{n\to\infty}N(n)\leq S_{0}$. Therefore, $(S(n),E(n),I(n),R(n))$ is ultimately bounded. This completes the proof. □

### Remark 2.2

From the previous discussion we see that the region

$$\Gamma=\bigl\{ (S,E,I,R): S\geq0, E\geq0, I\geq0, R\geq0, S+E+I+R\leq S_{0}\bigr\} $$

is a positive invariable set for model (2) and absorbs all nonnegative solutions of model (2). Therefore, we can assume in the rest of this paper that $S(n)\leq S_{0}$, $E(n)\leq S_{0}$, $I(n)\leq S_{0}$, and $R(n)\leq S_{0}$ for all $n\geq0$.

The basic reproduction number for model (2) is given by

$$\mathcal{R}_{0}=\frac{\frac{\partial f}{\partial I}(S_{0},0,0,0)\delta}{(\mu _{3}+\gamma)(\mu_{2}+\delta)}. $$

Particularly, when $f(S,E,I,R)=\beta h(S)g(I)$ and $f(S,E,I,R)=\beta \frac{SI}{N}$, $\mathcal{R}_{0}$ becomes of the following forms, respectively,

$$\mathcal{R}_{0}=\frac{\beta h(S_{0})g'(0)\delta}{(\mu_{3}+\gamma)(\mu_{2}+\delta )}, \qquad\mathcal{R}_{0}= \frac{\beta\delta}{(\mu_{3}+\gamma)(\mu_{2}+\delta)}. $$

On the existence of equilibria of model (2), we have the following result.

### Theorem 2.2

1. *If* $\mathcal{R}_{0}\leq1$, *then model* (2) *has only a disease*-*free equilibrium* $P_{0}(S_{0},0,0,0)$, *where* $S_{0}=\frac{\Lambda}{\mu_{1}}$.
2. *If* $\mathcal{R}_{0}>1$, *then model* (2) *has a unique endemic equilibrium* $P_{*} (S_{*},E_{*},I_{*},R_{*})$, *except for* $P_{0}$.

### Proof

Any equilibrium $(S,E,I,R)$ of model (2) satisfies the equations

6 $$ \left \{ \textstyle\begin{array}{@{}l} \Lambda-f(S,E,I,R)-\mu_{1}S+\sigma R=0,\\ f(S,E,I,R)-(\mu_{2}+\delta)E=0,\\ \delta E-(\mu_{3}+\gamma)I=0,\\ \gamma I-(\mu_{4}+\sigma)R=0. \end{array}\displaystyle \right . $$

Hence, we have

$$E=\frac{\mu_{3}+\gamma}{\delta}I\triangleq E(I),\qquad R=\frac{\gamma}{\mu _{4}+\sigma}I\triangleq R(I), $$

and

$$\Lambda-(\mu_{2}+\delta)E-\mu_{1}S+\sigma R=\Lambda- \frac{(\mu_{2}+\delta)(\mu _{3}+\gamma)}{\delta}I-\mu_{1}S+\frac{\sigma\gamma}{\mu_{4}+\sigma}I=0. $$

Thus,

$$S=\frac{1}{\mu_{1}} \biggl[\Lambda-\frac{(\mu_{2}+\delta)(\mu_{3}+\gamma)(\mu _{4}+\sigma) -\delta\gamma\sigma}{\delta(\mu_{4}+\sigma)}I \biggr]\triangleq S(I). $$

Let $I^{*}=\frac{\Lambda\delta(\mu_{4}+\sigma)}{(\mu_{2}+\delta)(\mu_{3}+\gamma)(\mu _{4}+\sigma)-\delta\gamma\sigma}$. Then $I^{*}>0$, $S(I^{*})=0$, and $S(I)$ is decreasing for $I\in[0,\infty)$. From the second equation of (6) we have

$$f\bigl(S(I),E(I),I,R(I)\bigr)-\frac{(\mu_{2}+\delta)(\mu_{3}+\gamma)}{\delta}I=0. $$

Define

$$\Phi(I)=\frac{f(S(I),E(I),I,R(I))}{I}-\frac{(\mu_{2}+\delta)(\mu_{3}+\gamma )}{\delta}. $$

By (H), $\Phi(I)$ is decreasing for $I>0$, $\Phi(I^{*})=-\frac{(\mu _{2}+\delta)(\mu_{3}+\gamma)}{\delta}<0$, and

$$\lim_{I\to0^{+}}\Phi(I)=\frac{\partial f(S_{0},0,0,0)}{\partial I}-\frac {(\mu_{2}+\delta)(\mu_{3}+\gamma)}{\delta}. $$

If $\mathcal{R}_{0}\leq1$, then $\lim_{I\to0^{+}}\Phi(I)\leq0$. Hence, $\Phi(I)=0$ has no positive roots. This shows that model (2) has only a disease-free equilibrium $P_{0}$.

If $\mathcal{R}_{0}>1$, then $\lim_{I\to0^{+}}\Phi(I)>0$. Hence, $\Phi(I)=0$ has a unique positive root $I_{*}$. This shows that model (2) has a unique endemic equilibrium $P_{*}(S_{*},E_{*},I_{*},R_{*})$, where

$$S_{*}=\frac{1}{\mu_{1}} \biggl[\Lambda-\frac{(\mu_{2}+\delta)(\mu_{3}+\gamma)(\mu _{4}+\sigma) -\delta\gamma\sigma}{\delta(\mu_{4}+\sigma)}I_{*} \biggr] $$

and

$$E_{*}=\frac{\mu_{3}+\gamma}{\delta}I_{*},\qquad R_{*}=\frac{\gamma}{\mu_{4}+\sigma}I_{*}. $$

This completes the proof. □

We have the following result on the local stability of the disease-free equilibrium and endemic equilibrium.

### Theorem 2.3

*When* $\mathcal{R}_{0}<1$, *the disease*-*free equilibrium* $P_{0}$ *of model* (2) *is locally asymptotically stable*, *and when* $\mathcal {R}_{0}>1$, $P_{0}$ *is unstable*.

### Proof

The linearization system of model (2) at equilibrium $P_{0}$ is

7 $$ \left \{ \textstyle\begin{array}{@{}l} x_{n+1}= x_{n}-\frac{\partial f}{\partial I}(S_{0},0,0,0)z_{n+1}-\mu_{1}x_{n+1}+\sigma u_{n+1},\\ y_{n+1}= y_{n}+\frac{\partial f}{\partial I}(S_{0},0,0,0)z_{n+1}-(\mu_{2}+\delta)y_{n+1},\\ z_{n+1}= z_{n}+\delta y_{n+1}-(\mu_{3}+\gamma)z_{n+1},\\ u_{n+1}= u_{n}+\gamma z_{n+1}-(\mu_{4}+\sigma)u_{n+1}. \end{array}\displaystyle \right . $$

From the second and third equations of system (7) we have

8 $$ \begin{pmatrix} y_{n+1}\\ z_{n+1} \end{pmatrix} =A^{-1} \begin{pmatrix} y_{n}\\ z_{n} \end{pmatrix}, $$

where

$$A= \begin{pmatrix} 1+\mu_{2}+\delta& -\frac{\partial f}{\partial I}(S_{0},0,0,0)\\ -\delta& 1+\mu_{3}+\gamma \end{pmatrix}. $$

Since $\mathcal{R}_{0}<1$, we easily prove that two eigenvalues $\lambda _{i} $ ($i=1,2$) of the matrix *A* satisfy $|\lambda_{i}|>1$. Therefore, two eigenvalues $\rho_{i}$ ($i=1,2$) of the matrix $A^{-1}$ satisfy $|\rho_{i}|<1$.

From the first and fourth equations of system (7) we have

$$\begin{pmatrix} x_{n+1}\\ u_{n+1} \end{pmatrix} =B^{-1} \begin{pmatrix} x_{n}\\ u_{n} \end{pmatrix} +B^{-1} \begin{pmatrix} -\frac{\partial f}{\partial I}(S_{0},0,0,0)\\ \gamma \end{pmatrix} y_{n+1}, $$

where

$$B= \begin{pmatrix} 1+\mu_{1} & -\delta\\ 0 & 1+\mu_{4}+\sigma \end{pmatrix}. $$

Obviously, the matrix $B^{-1}$ has eigenvalues $\rho_{i}$ ($i=3,4$) satisfying $|\rho_{i}|<1$. Therefore, equilibrium $(0,0,0,0)$ of system (7) is asymptotically stable. Consequently, when $\mathcal {R}_{0}<1$, the equilibrium $P_{0}$ of model (2) is locally asymptotically stable.

When $\mathcal{R}_{0}>1$, we easily prove that two eigenvalues $\rho_{i} $ ($i=1,2$) of the matrix $A^{-1}$ are real numbers and $|\rho_{1}|<1$ and $|\rho_{2}|>1$. Hence, the equilibrium $(0,0)$ of system (8) is unstable. This shows that the equilibrium $P_{0}$ is unstable when $\mathcal {R}_{0}>1$. □

### Remark 2.3

It is unfortunate that we do not establish the local asymptotic stability of endemic equilibrium $P_{*}$ of model (2). In fact, the linearization system of model (2) at endemic equilibrium $P_{*}$ is

$$\begin{pmatrix} x_{n+1}\\ y_{n+1}\\ z_{n+1}\\ u_{n+1} \end{pmatrix} =C^{-1} \begin{pmatrix} x_{n}\\ y_{n}\\ z_{n}\\ u_{n} \end{pmatrix}, $$

where

$$C= \begin{pmatrix} 1+\frac{\partial f}{\partial S}(S_{*},E_{*},I_{*},R_{*})+\mu_{1} & 0 & \frac{\partial f}{\partial I}(S_{*},E_{*},I_{*},R_{*}) & -\sigma\\ -\frac{\partial f}{\partial S}(S_{*},E_{*},I_{*},R_{*}) & 1+\mu _{2}+\delta& -\frac{\partial f}{\partial I}(S_{*},E_{*},I_{*},R_{*}) & 0\\ 0 & -\delta& 1+\mu_{3}+\gamma& 0\\ 0 & 0 & -\gamma& 1+\mu_{4}+\sigma \end{pmatrix}. $$

In order to obtain the local asymptotic stability of endemic equilibrium $P_{*}$, we only need to prove that all eigenvalues *λ* of matrix $C^{-1}$ satisfy $|\lambda|<1$. However, it is a pity that here we do not obtain this.

Therefore, when $\mathcal{R}_{0}>1$, whether the endemic equilibrium $P_{*}$ of model (2) also is locally asymptotically stable still is an interesting open problem.

## Global attractivity of disease-free equilibrium

In this section, we discuss the global attractivity of disease-free equilibrium of model (2). We have the following result.

### Theorem 3.1

*The disease*-*free equilibrium* $P_{0}$ *of model* (2) *is globally attractive if and only if* $\mathcal{R}_{0}\leq1$.

### Proof

The necessity is obvious because when $\mathcal{R}_{0}> 1$, model (2) has an endemic equilibrium $P_{*}$. Now, we prove the sufficiency. When $\mathcal{R}_{0}\leq1$, we can choose a constant $p>0$ such that

9 $$ \frac{\delta}{p}-(\mu_{2}+\delta)\leq0,\qquad \frac{\partial f}{\partial I}(S_{0},0,0,0)p-( \mu_{3}+\gamma)\leq0. $$

Let $(S(n),E(n),I(n),R(n))$ be any positive solution of model (2). Choosing the Lyapunov function

$$V(n)=pE(n)+I(n), $$

we have

$$\begin{aligned} \triangle V(n)={}& V(n+1)-V(n) \\ ={}& p\bigl(f\bigl(S(n+1),E(n+1),I(n+1),R(n+1)\bigr)-(\mu_{2}+ \delta)E(n+1)\bigr) \\ &{}+\bigl(\delta E(n+1)-(\mu_{3}+\gamma)I(n+1)\bigr) \\ < {}& p\biggl(\frac{\partial f(S_{0},0,0,0)}{\partial I}I(n+1)-(\mu _{2}+\delta)E(n+1)\biggr) \\ &{}+\bigl(\delta E(n+1)-(\mu_{3}+\gamma)I(n+1)\bigr) \\ ={}& \biggl[p\frac{\partial f(S_{0},0,0,0)}{\partial I}-(\mu_{3}+\gamma )\biggr]I(n+1)+ \biggl[\frac{\delta}{p}-(\mu_{2}+\delta)\biggr]pE(n+1). \end{aligned}$$

From (9) we have $\triangle V(n)\leq0$. It is clear that $\{(S,E,I,R): \triangle V(n)=0\}\subset\{(S,E,I,R): I=0\}$. When $I(n)\equiv0$, from the third equation of model (2) we have $E(n)\equiv0$. From the fourth equation of model (2) we further have $\lim_{n\to\infty}R(n)=0$. From the first equation of model (2) we also have $\lim_{n\to\infty}S(n)=S_{0}$. This shows that the maximal invariable set in $\{(S,E,I,R): \triangle V(n)=0\}$ is a disease-free equilibrium $P_{0}$.

Therefore, using the theorems of stability of difference equations (see Theorem 6.3 in [37]), we finally obtain that the disease-free equilibrium $P_{0}$ of model (2) is globally attractive. This completes the proof. □

### Remark 3.1

When $f(S,E,I,R)=\frac{SI}{N}$ (standard incidence), by Theorem 3.1, if $\mathcal{R}_{0}= \frac{\beta\delta}{(\mu_{3}+\gamma)(\mu_{2}+\delta)}\leq 1$, then the disease-free equilibrium $P_{0}$ in model (2) is globally attractive.

## Permanence of disease

For model (2), disease $I(n)$ is said to be permanent if there exists constants $M>m>0$ such that for any solution $(S(n),E(n),I(n),R(n))$ of model (2) with initial condition (3), $m\leq\liminf_{n\rightarrow\infty}I(n) \leq\limsup_{n\rightarrow\infty}I(n)\leq M$. We have the following result.

### Theorem 4.1

*Disease* $I(n)$ *in model* (2) *is permanent if and only if* $\mathcal {R}_{0}>1$.

### Proof

The necessity is obvious. In fact, if $\mathcal{R}_{0}\leq1$, then by Theorem 3.1 the disease-free equilibrium $P_{0}$ is globally attractive.

Now, we prove the sufficiency. When $\mathcal{R}_{0}>1$, we can choose constants $p>0$ and $\varepsilon_{0}>0$ such that

10 $$ \frac{\delta}{p}-(\mu_{2}+\delta)>0,\qquad \biggl(\frac{\partial f}{\partial I}(S_{0},0,0,0)- \varepsilon_{0}\biggr)p-(\mu_{3}+\gamma)>0. $$

We will use the persistence theory of dynamical systems (see [35], Section 1.3 in Chapter 1) to prove the theorem. Define the sets

$$X=\bigl\{ (S,E,I,R): S>0,E\geq0,I\geq0,R\geq0\bigr\} $$

and

$$X_{0}=\bigl\{ (S,E,I,R)\in X: E>0,I>0\bigr\} ,\qquad \partial X_{0}=\bigl\{ (S,E,I,R)\in X: EI=0\bigr\} . $$

Let $(S(n),E(n),I(n),R(n))$ be the solution of model (2) with initial condition $(S(0),E(0), I(0),R(0))=(S_{0},E_{0},I_{0},R_{0})$. Define the set

$$M_{\partial}=\bigl\{ (S_{0},E_{0},I_{0},R_{0}) \in\partial X_{0}: \bigl(S(n),E(n),I(n),R(n)\bigr)\in\partial X_{0}, n=1,2,\ldots\bigr\} . $$

It is clear that the solution of model (2) with initial condition $(S(0),E(0),I(0),R(0))=(S_{0},0,0,R_{0})$ has the form $(S(n),0,0,R(n))$. Hence, we have

$$\bigl\{ (S_{0},0,0,R_{0}): S_{0}>0,R_{0} \geq0\bigr\} \subset M_{\partial}. $$

Suppose that there is $(S_{0},E_{0},I_{0},R_{0})\in M_{\partial}$ such that $(S_{0},E_{0},I_{0},R_{0})\notin\{(S_{0},0,0,R_{0}): S_{0}>0,R_{0}\geq0\}$. Then, we have $E_{0}>0$ or $I_{0}>0$. Let $(S(n),E(n),I(n),R(n))$ be the solution of model (2) with initial condition $(S(0),E(0),I(0),R(0))=(S_{0},E_{0},I_{0},R_{0})$. If $E_{0}>0$, then from the second equation of model (2) we have

$$E(n+1)\geq E(n)-(\mu_{2}+\delta)E(n+1). $$

Hence, $E(n)\geq E(0)(\frac{1}{1+\mu_{2}+\delta})^{n}>0$ for all $n\geq 0$. From the third equation of model (2) we further have

$$I(n+1)> I(n)-(\mu_{3}+\gamma)I(n+1), \quad n\geq0. $$

Hence, $I(n)>I(0)(\frac{1}{1+\mu_{3}+\gamma})^{n}\geq0$ for all $n\geq0$. This shows that a solution $(S(n),E(n),I(n), R(n))\notin\partial X_{0}$ for all $n>0$. If $I_{0}>0$, then from third equation of model (2) we have $I(n)\geq I(0)(\frac{1}{1+\mu_{3}+\gamma})^{n}> 0$ for all $n\geq0$. Since $S(n)>0$ and $f(S(n),I(n))>0$ for all $n\geq0$, from the second equation of model (2) we further have $E(n)> E(0)(\frac{1}{1+\mu_{2}+\delta})^{n}\geq0$ for all $n\geq0$. This also shows that the solution $(S(n),E(n),I(n),R(n))\notin\partial X_{0}$ for all $n>0$. Hence, $(S_{0},E_{0},I_{0},R_{0})\notin M_{\partial}$, which leads to a contradiction. Thus, we also have

$$M_{\partial}\subset\bigl\{ (S_{0},0,0,R_{0}): S_{0}>0,R_{0}\geq0\bigr\} . $$

Therefore, $M_{\partial}=\{(S_{0},0,0,R_{0}): S_{0}>0,R_{0}\geq0\}$.

It is clear that model (2) restricted to $M_{\partial}$ has a globally attractive equilibrium $P_{0}(S_{0}, 0,0,0)$. This shows that $\{P_{0}\}$ in $M_{\partial}$ is isolated invariable and acyclic. Now, we prove that

$$W^{s}(P_{0})\cap X_{0}=\emptyset, $$

where

$$W^{s}(P_{0})=\Bigl\{ \bigl(S(0),E(0),I(0),R(0)\bigr): \lim _{n\to\infty }\bigl(S(n),E(n),I(n),R(n)\bigr)=P_{0}\Bigr\} , $$

which is said to be a stable set of $P_{0}$. Suppose that there is a point $(S(0),E(0),I(0),R(0))\in X_{0}$ such that $\lim_{n\to\infty }(S(n),E(n),I(n),R(n))=P_{0}$. Since

$$\lim_{(S,E,I,R)\to P_{0}}\frac{f(S,E,I,R)}{I}=\frac{\partial f(S_{0},0,0,0)}{\partial I}, $$

for the above $\varepsilon_{0}>0$, there is $\eta_{0}>0$ such that when $|S-S_{0}|<\eta_{0}$, $E<\eta_{0}$, $I<\eta_{0}$, and $R<\eta_{0}$, we have

$$\frac{f(S,E,I,R)}{I}\geq\frac{\partial f(S_{0},0,0,0)}{\partial I}-\varepsilon_{0}. $$

We can choose an integer $n_{0}>0$ such that $|S(n)-S_{0}|<\eta_{0}$, $E(n)<\eta_{0}$, $I(n)<\eta_{0}$, and $R(n)<\eta_{0}$ for all $n\geq n_{0}$.

Consider the Lyapunov function

$$V(n)=pE(n)+I(n). $$

We have that, for $n>n_{0}$,

$$\begin{aligned} \triangle V(n)={}& V(n+1)-V(n) \\ ={}& p\bigl(f\bigl(S(n+1),E(n+1),I(n+1),R(n+1)\bigr)-(\mu_{2}+ \delta)E(n+1)\bigr) \\ &{}+\bigl(\delta E(n+1)-(\mu_{3}+\gamma)I(n+1)\bigr) \\ \geq{}& p\biggl(\biggl(\frac{\partial f(S_{0},0,0,0)}{\partial I}-\varepsilon_{0} \biggr)I(n+1)-(\mu_{2}+\delta)E(n+1)\biggr) \\ &{}+\bigl(\delta E(n+1)-(\mu_{3}+\gamma)I(n+1)\bigr) \\ ={}& \biggl[p\biggl(\frac{\partial f(S_{0},0,0,0)}{\partial I}-\varepsilon _{0}\biggr)-( \mu_{3}+\gamma)\biggr]I(n+1)+\biggl[\frac{\delta}{p}-( \mu_{2}+\delta)\biggr]pE(n+1) \\ \geq{}& mV(n+1), \end{aligned}$$

where

$$m=\min\biggl\{ p\biggl(\frac{\partial f(S_{0},0,0,0)}{\partial I}-\varepsilon_{0}\biggr)-(\mu _{3}+\gamma),\frac{\delta}{p}-(\mu_{2}+\delta)\biggr\} >0. $$

Hence, we finally have $\lim_{n\to\infty}V(n)=\infty$, which leads to a contradiction with $\lim_{n\to\infty}V(n)=0$. It follows that $W^{s}(P_{0})\cap X_{0}=\emptyset$. Thus, by the theorems of uniform persistence for dynamical systems given in [38], we obtain that model (2) is permanent. This completes the proof. □

### Remark 4.1

When $f(S,E,I,R)=\frac{SI}{N}$, by Theorem 4.1, if $\mathcal{R}_{0}=\frac{\beta\delta}{(\mu_{3}+\gamma)(\mu_{2}+\delta)}>1$, then the disease in model (2) is permanent.

### Remark 4.2

Theorem 4.1 only obtains the permanence of the disease for model (2). However, whether we can also prove that an endemic equilibrium $P_{*}$ is globally attractive for model (2) when $\mathcal{R}_{0}>1$? In the following section, we will give a partial positive answer. We will prove that, for special case $\sigma=0$ of model (2), an endemic equilibrium $P_{*}$ is globally attractive only when $\mathcal{R}_{0}>1$.

## Global attractivity of endemic equilibrium in a particular case

In this section, we consider a particular case of model (2), that is, $f(S,E,I,R)=f(S,E,I)$ and $\sigma=0$ in model (2). Model (2) becomes of the form

11 $$ \left \{ \textstyle\begin{array}{@{}l} S(n+1)= S(n)+\Lambda-f(S(n+1),E(n+1),I(n+1)) -\mu_{1} S(n+1),\\ E(n+1)= E(n)+f(S(n+1),E(n+1),I(n+1))-(\mu_{2}+\delta )E(n+1),\\ I(n+1)= I(n)+\delta E(n+1)-(\mu_{3}+\gamma)I(n+1),\\ R(n+1)= R(n)+\gamma I(n+1)-\mu_{4}R(n+1). \end{array}\displaystyle \right . $$

Because $R(n)$ does not appear in the first three equations of model (2), we only need to consider the equivalent system

12 $$ \left \{ \textstyle\begin{array}{@{}l} S(n+1)= S(n)+\Lambda-f(S(n+1),E(n+1),I(n+1)) -\mu_{1} S(n+1),\\ E(n+1)= E(n)+f(S(n+1),E(n+1),I(n+1))-(\mu_{2}+\delta )E(n+1),\\ I(n+1)= I(n)+\delta E(n+1)-(\mu_{3}+\gamma)I(n+1). \end{array}\displaystyle \right . $$

We have the following result on the global attractivity of the endemic equilibrium for model (12).

### Theorem 5.1

*If* $\mathcal{R}_{0}>1$, *then the endemic equilibrium* $P_{*}$ *of model* (12) *is globally attractive*.

### Proof

Let $P_{*}(S_{*},E_{*},I_{*})$ be an endemic equilibrium of model (12). Then

13 $$ \left \{ \textstyle\begin{array}{@{}l} \Lambda-f(S_{*},E_{*},I_{*}) -\mu_{1} S_{*}=0,\\ f(S_{*},E_{*},I_{*})-(\mu_{2}+\delta)E_{*}=0,\\ \delta E_{*}-(\mu_{3}+\gamma)I_{*}=0. \end{array}\displaystyle \right . $$

Let $(S(n),E(n),I(n))$ be any positive solution of system (12). Define the functions

$$\begin{aligned}& V_{1}\bigl(S(n)\bigr)=S(n)-S_{*}- \int^{S(n)}_{S_{*}}\frac{f(S_{*},E_{*},I_{*})}{f(\eta ,E_{*},I_{*})}\,d\eta, \\& V_{2}\bigl(E(n)\bigr)=E(n)-E_{*}-E_{*}\ln\frac{E(n)}{E_{*}}, \end{aligned}$$

and

$$V_{3}\bigl(I(n)\bigr)=I(n)-I_{*}-I_{*}\ln\frac{I(n)}{I_{*}}. $$

From (H) we easily obtain that when $S(n)\neq S_{*}$,

$$V_{1}\bigl(S(n)\bigr)>S(n)-S_{*}- \int_{S_{*}}^{S(n)}\frac{f(S_{*},E_{*},I_{*})}{f(S_{*},E_{*},I_{*})}\,d\eta=0. $$

Since $g(x)=x-1-\ln x>0$ for $x>0$ and $x\neq1$, we obtain that when $E(n)\neq E_{*}$ and $I(n)\neq I_{*}$, $V_{2}(E(n))>0$ and $V_{3}(I(n))>0$. Computing $\triangle V_{1}(n)=V_{1}(S(n+1))-V_{1}(S(n))$, we have

$$\triangle V_{1}(n)=S(n+1)-S(n)- \int^{S(n+1)}_{S(n)} \frac{f(S_{*},E_{*},I_{*})}{f(\eta,E_{*},I_{*})}\,d\eta. $$

From (H) it follows that, for any *η* between $S(n)$ and $S(n+1)$,

$$\begin{aligned}& -\frac{f(S_{*},E_{*},I_{*})}{f(\eta,E_{*},I_{*})}\leq -\frac{f(S_{*},E_{*},I_{*})}{f(S(n+1),E_{*},I_{*})} \quad \mbox{if } S(n+1)\geq S(n), \\& -\frac{f(S_{*},E_{*},I_{*})}{f(\eta,E_{*},I_{*})}\geq -\frac{f(S_{*},E_{*},I_{*})}{f(S(n+1),E_{*},I_{*})} \quad \mbox{if } S(n+1)\leq S(n). \end{aligned}$$

We have

$$- \int^{S(n+1)}_{S(n)} \frac{f(S_{*},E_{*},I_{*})}{f(\eta,E_{*},I_{*})}\,d\eta\leq - \frac{f(S_{*},E_{*},I_{*})}{f(S(n+1),E_{*},I_{*})}\bigl(S(n+1)-S(n)\bigr). $$

Therefore, from (13) we obtain

14 $$\begin{aligned} \triangle V_{1}(n)\leq{}& \biggl[1-\frac{f(S_{*},E_{*},I_{*})}{f(S(n+1),E_{*},I_{*})} \biggr]\bigl(S(n+1)-S(n)\bigr) \\ ={}& \biggl[1-\frac{f(S_{*},E_{*},I_{*})}{f(S(n+1),E_{*},I_{*})} \biggr] \bigl[\Lambda-f\bigl(S(n+1),E(n+1),I(n+1) \bigr) \\ &{}-\mu_{1} S(n+1) \bigr] \\ ={}& -\mu_{1} \biggl[1-\frac {f(S_{*},E_{*},I_{*})}{f(S(n+1),E_{*},I_{*})} \biggr] \bigl(S(n+1)-S_{*} \bigr) \\ &{} +f(S_{*},E_{*},I_{*})-f\bigl(S(n+1),E(n+1),I(n+1)\bigr) \\ &{} -\frac{f(S_{*},E_{*},I_{*})}{f(S(n+1),E_{*},I_{*})}f(S_{*},E_{*},I_{*}) \\ &{} +\frac{f(S_{*},E_{*},I_{*})}{f(S(n+1),E_{*},I_{*})}f\bigl(S(n+1),E(n+1),I(n+1)\bigr). \end{aligned}$$

Calculating $\Delta V_{2}(n)=V_{2}(E(n+1))-V_{2}(E(n))$, we obtain

$$\Delta V_{2}(n) =E(n+1)-E(n)-I_{*}\ln\frac{E(n+1)}{E(n)}. $$

Using the inequality $\ln(1-x)\leq-x$ for $x<1$, we have

$$-\ln\frac{E(n+1)}{E(n)}=\ln \biggl[1-\biggl(1-\frac{E(n)}{E(n+1)}\biggr) \biggr] \leq - \biggl[1-\frac{E(n)}{E(n+1)} \biggr]. $$

Therefore,

15 $$\begin{aligned} \triangle V_{2}(n)\leq{}& \biggl[1-\frac{E_{*}}{E(n+1)} \biggr]\bigl(E(n+1)-E(n)\bigr) \\ ={}& \biggl[1-\frac{E_{*}}{E(n+1)} \biggr] \bigl[f\bigl(S(n+1),E(n+1),I(n+1)\bigr) -(k+\mu_{2})E(n+1) \bigr] \\ ={}& f\bigl(S(n+1),E(n+1),I(n+1)\bigr)-(k+\mu_{2})E(n+1) \\ &{} -\frac{E_{*}}{E(n+1)}f\bigl(S(n+1),E(n+1),I(n+1)\bigr)+(k+ \mu_{2})E_{*}. \end{aligned}$$

Similarly, calculating $\Delta V_{3}(n)=V_{3}(I(n+1))-V_{2}(I(n))$, we obtain

16 $$\begin{aligned} \triangle V_{3}(n)\leq{}& \delta E(n+1)-( \mu_{3}+\gamma)I(n+1) \\ &{} -\frac{I_{*}}{I(n+1)}\delta E(n+1)+(\mu_{3}+\gamma)I_{*}. \end{aligned}$$

Choose the Lyapunov function

$$V(n)=V_{1}\bigl(S(n)\bigr)+V_{2}\bigl(E(n)\bigr)+ \frac{f(S_{*},E_{*},I_{*})}{(\mu_{3}+\gamma)I_{*}} V_{3}\bigl(I(n)\bigr). $$

For convenience of calculations, we denote $S=S(n+1)$, $E=E(n+1)$, and $I=I(n+1)$. Computing $\triangle V(n)=V(n+1)-V(n)$, from (14)-(16) we obtain

$$\begin{aligned} \triangle V(n)\leq{}& {-}\mu_{1} \biggl[1-\frac {f(S_{*},E_{*},I_{*})}{f(S,E_{*},I_{*})} \biggr](S-S_{*}) +f(S_{*},E_{*},I_{*}) \biggl[3-\frac{f(S_{*},E_{*},I_{*})}{f(S,E_{*},I_{*})} \\ &{} +\frac{f(S,E,I)}{f(S,E_{*},I_{*})}-\frac{E^{*}f(S,E,I)}{Ef(S_{*},E_{*},I_{*})} -\frac{I}{I_{*}}- \frac{I_{*}E}{IE^{*}} \biggr] \\ ={}& f(S_{*},E_{*},I_{*}) \biggl[1-\frac{I_{*}E}{IE^{*}}+\ln\frac {I_{*}E}{IE^{*}} \biggr] -f(S_{*},E_{*},I_{*})\ln\frac{I_{*}E}{IE^{*}} \\ &{} +f(S_{*},E_{*},I_{*}) \biggl[1-\frac{f(S,E,I)E^{*}}{f(S_{*},E_{*},I_{*})E} +\ln\frac{f(S,E,I)E^{*}}{f(S_{*},E_{*},I_{*})E} \biggr] \\ &{} -f(S_{*},E_{*},I_{*})\ln\frac{f(S,E,I)E^{*}}{f(S_{*},E_{*},I_{*})E} \\ &{} +f(S_{*},E_{*},I_{*}) \biggl[1-\frac{f(S_{*},E_{*},I_{*})}{f(S,E_{*},I_{*})} +\ln\frac{f(S_{*},E_{*},I_{*})}{f(S,E_{*},I_{*})} \biggr] \\ &{} -f(S_{*},E_{*},I_{*})\ln\frac{f(S_{*},E_{*},I_{*})}{f(S,E_{*},I_{*})} \\ &{} +\frac{f(S_{*},E_{*},I_{*})f(S,E,I)}{f(S,E_{*},I_{*})} \biggl[1-\frac{If(S,E_{*},I_{*})}{I_{*}f(S,E,I)} +\ln\frac{If(S,E_{*},I_{*})}{I_{*}f(S,E,I)} \biggr] \\ &{} -\frac{f(S_{*},E_{*},I_{*})f(S,E,I)}{f(S,E_{*},I_{*})} \ln\frac{If(S,E_{*},I_{*})}{I_{*}f(S,E,I)} \\ \leq{}& {-}f(S_{*},E_{*},I_{*})\ln\frac{I_{*}f(S,E,I)}{If(S,E_{*},I_{*})} -\frac{f(S_{*},E_{*},I_{*})f(S,E,I)}{f(S,E_{*},I_{*})}\ln \frac {If(S,E_{*},I_{*})}{I_{*}f(S,E,I)} \\ ={}& \frac{f(S_{*},E_{*},I_{*})}{f(S,E,I)} \bigl[f(S,E_{*},I_{*})-f(S,E,I) \bigr] \biggl[\ln \frac{f(S,E_{*},I_{*})}{I_{*}}-\ln\frac{f(S,E,I)}{I} \biggr]. \end{aligned}$$

From (H) we obtain $\triangle V(n)\leq0$ for any $n\geq0$, and $\triangle V(n)\equiv0$ implies $I(n)\equiv I_{*}$ for all $n\geq 0$. From $I(n)\equiv I_{*}$ and the third equation of model (12) it follows that $E(n)\equiv E_{*}$ for all $n\geq0$. Furthermore, from the second equation of model (12) we obtain that $S(n)\equiv S_{*}$ for all $n\geq0$.

Therefore, using the theorems of stability of difference equations, we finally obtain that the endemic equilibrium $P_{*}$ of model (12) is globally attractive. This completes the proof. □

### Remark 5.1

In Remark 4.2, we indicated that for SEIRS-type model (2), an important problem is to prove that the endemic equilibrium is globally attractive only when $\mathcal{R}_{0}>1$. From Theorem 5.1 we see that only for the particular case $\sigma=0$ of model (2), that is, SEIR-type model, we get a positive answer. Therefore, an interesting open problem for general SEIRS model (2) is whether the endemic equilibrium is also globally attractive only when $\mathcal{R}_{0}>1$.

### Remark 5.2

From the proofs of Theorem 3.1, Theorem 4.1, and Theorem 5.1 we easily see that the condition $\mu_{1}\leq\min\{\mu_{2},\mu_{3},\mu_{4}\}$ is not used. In fact, this condition is only used in Theorem 2.1 to obtain the positivity of solutions of model (2). Therefore, an interesting question is whether the condition $\mu_{1}\leq\min\{\mu_{2},\mu_{3},\mu_{4}\}$ can be taken out in the proof of the positivity of solutions of model (2).

## Numerical examples

Now, we give numerical examples to show that for SEIRS-type model (2), the endemic equilibrium may be globally attractive for different incidence function $f(S,E,I,R)$, which satisfies (H) only when the basic reproduction number $\mathcal{R}_{0}>1$.

### Example 6.1

In model (2), we take $f(S,E,I,R)=\frac{\beta SI}{1+\alpha I+\omega S}$, $\Lambda=1.5$, $\mu_{1}=0.2$, $\mu_{2}=0.35$, $\mu_{3}=0.5$, $\beta=0.36$, $\delta=0.3$, $\omega=0.1$, and $\gamma=0.1$. The parameters $\mu_{4}$, *α*, and *σ* will be chosen later.

**Figure 1:**
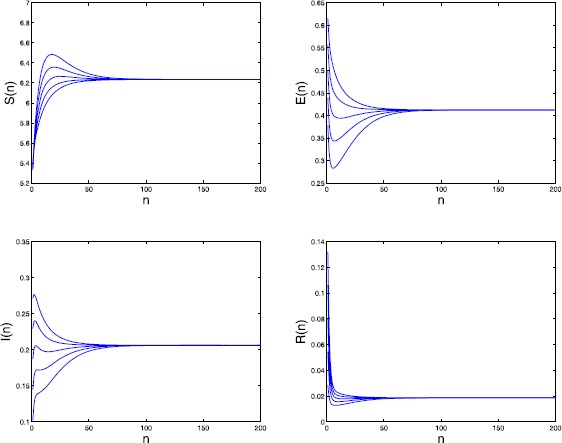
**Time series of** $\pmb{S(n)}$ **,** $\pmb{E(n)}$ **,** $\pmb{I(n)}$ **, and** $\pmb{R(n)}$ **in Example** **6.1**

By calculating we have the basic reproduction number $\mathcal{R}_{0}=1.1868>1$. We further take $\mu_{4}=0.3$, $\alpha=0.5$, and $\sigma=0.8$. Then the endemic equilibrium $P_{*}=(6.235,0.412,0.206, 0.019)$. From the numerical simulations (see Figure 1) we obtain that $P_{*}$ may be globally attractive.

### Example 6.2

In model (2), we take $f(S,E,I,R)=\frac{\beta SI}{1+\alpha I^{2}}$, $\Lambda=2.5$, $\mu_{1}=0.2$, $\mu_{2}=0.35$, $\mu_{3}=0.5$, $\beta=0.3$, $\delta=0.4$, and $\gamma=0.6$. The parameters $\mu_{4}$, *α*, and *σ* will be chosen later.

**Figure 2:**
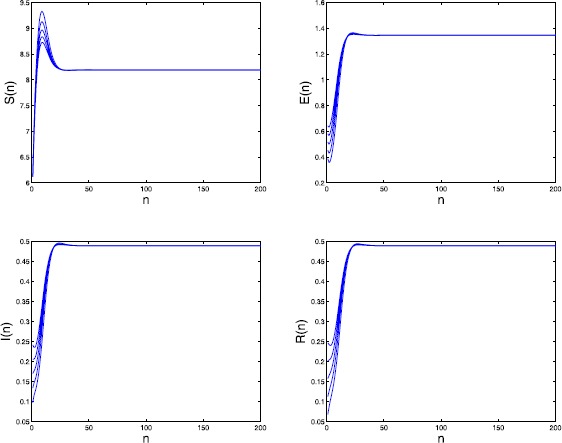
**Time series of** $\pmb{S(n)}$ **,** $\pmb{E(n)}$ **,** $\pmb{I(n)}$ **, and** $\pmb{R(n)}$ **in Example** **6.2**

By calculating we have the basic reproduction number $\mathcal{R}_{0}=1.8182>1$. We further take $\mu_{4}=0.3$, $\alpha=0.8$, and $\sigma=0.3$. Then the endemic equilibrium $P_{*}=(8.190,1.345,0.489, 0.489)$. By numerical simulations (see Figure 2) we obtain that $P_{*}$ may be globally attractive.

### Example 6.3

In model (2), we take $f(S,E,I,R)=\frac{\beta S^{2}I}{(1+\omega S)(1+\alpha I)}$, $\Lambda=5$, $\mu_{1}=0.9$, $\mu_{2}=0.6$, $\mu_{3}=0.5$, $\beta=0.3$, $\delta=0.2$, $\omega=0.3$, and $\gamma=0.3$. The parameters $\mu_{4}$, *α*, and *σ* will be chosen later.

**Figure 3:**
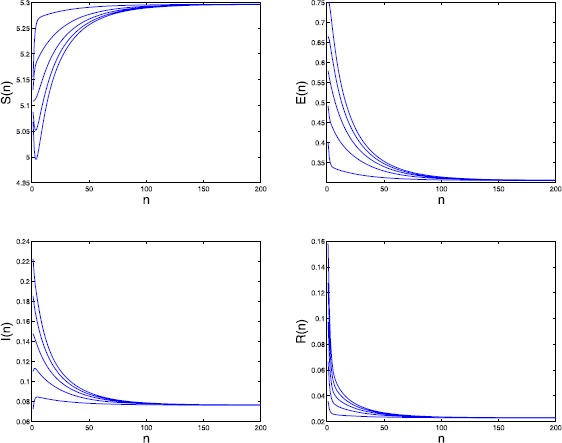
**Time series of** $\pmb{S(n)}$ **,** $\pmb{E(n)}$ **,** $\pmb{I(n)}$ **, and** $\pmb{R(n)}$ **in Example** **6.3**

By calculating we have the basic reproduction number $\mathcal{R}_{0}=1.0851>1$. We further take $\mu_{4}=0.5$, $\alpha=0.207$, and $\sigma=0.5$. Then the endemic equilibrium $P_{*}=(5.296,0.306,0.077, 0.023)$. By numerical simulations (see Figure 3) we obtain that $P_{*}$ may be globally attractive.

### Example 6.4

In model (2), we take $f(S,E,I,R)=\frac{\beta S^{2}I}{(1+\omega S)(1+\alpha I^{2})}$, $\Lambda=1.5$, $\mu_{1}=0.2$, $\mu_{2}=0.35$, $\mu_{3}=0.5$, $\beta=0.32$, $\delta=0.4$, $\omega=0.3$, and $\gamma=0.6$. The parameters $\mu_{4}$, *α*, and *σ* will be chosen later.

**Figure 4:**
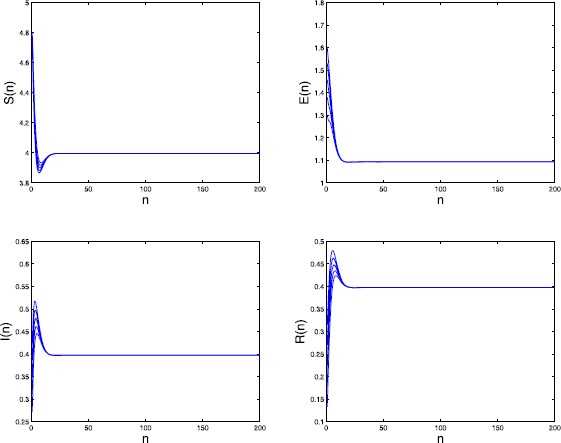
**Time series of** $\pmb{S(n)}$ **,** $\pmb{E(n)}$ **,** $\pmb{I(n)}$ **, and** $\pmb{R(n)}$ **in Example** **6.4**

By calculating we have the basic reproduction number $\mathcal{R}_{0}=2.6853>1$. We further take $\mu_{4}=0.3$, $\alpha=0.8$, and $\sigma=0.3$. Then the endemic equilibrium $P_{*}=(3.996,1.096,0.398, 0.398)$. By numerical simulations (see Figure 4) we obtain that $P_{*}$ may be globally attractive.

All these examples of numerical simulations show that when $\mathcal{R}_{0}>1$, no matter sufficiently greater than one or closer to one but still greater than one, we always obtain that the endemic equilibrium $P_{*}$ is globally attractive, which may offer an affirmative conjecture to the open problem given in Remark 5.1, that is, for the general SEIRS model (2) the endemic equilibrium $P_{*}$ is globally attractive only when $\mathcal{R}_{0}>1$. Therefore, in our future work, we expect to obtain the corresponding theoretical results for this open problem.

## Discussion

In this paper, we proposed a discrete SEIRS epidemic model (2) with general nonlinear incidence, which is described by the backward difference scheme. By our discussions presented in this paper, necessary and sufficient conditions for the global attractivity of the disease-free equilibrium and the permanence of the disease are established, that is, if the basic reproduction number $\mathcal {R}_{0}\leq1$, then the disease-free equilibrium is globally attractive, and if $\mathcal{R}_{0}> 1$, then the disease is permanent. Furthermore, when the model degenerates into SEIR model, it is proved that when $\mathcal{R}_{0}> 1$, the model has a unique globally attractive endemic equilibrium.

Unfortunately, for SEIRS model (2), when the basic reproduction number is greater than one, we do not obtain the local asymptotic stability and global attractivity of the endemic equilibrium. But the numerical examples given in Section 5 show that the endemic equilibrium for general SEIRS model (2) may be globally attractive. Therefore, it is still an important and interesting open problem how to apply the linearization method to establish the local asymptotic stability of the endemic equilibrium and how to construct the discrete analogue Lyapunov functions to study the global attractivity of the endemic equilibrium for general SEIRS model (2).

In addition, the dynamical behaviors for the nonautonomous discrete SEIRS epidemic models, discrete SEIRS epidemic models with vaccination, stage-structured discrete SEIRS epidemic models, and delayed discrete SEIRS epidemic models with nonlinear incidence described by the backward difference scheme are rarely considered. Whether similar results on the permanence and extinction of the disease and the global attractivity of the disease-free equilibrium for these models can be obtained is also an interesting open question.

On the other hand, corresponding to continuous model (1), we also have the following discrete SEIRS epidemic models with general nonlinear incidence described by the forward difference scheme or Micken’s nonstandard finite difference scheme:

$$\left \{ \textstyle\begin{array}{@{}l} S(n+1)-S(n)= \Lambda-f(X(n)) -\mu_{1} S(n)+\sigma R(n),\\ E(n+1)-E(n)= f(X(n))-(\mu_{2}+\delta)E(n),\\ I(n+1)-I(n)= \delta E(n)-(\mu_{3}+\gamma)I(n),\\ R(n+1)-R(n)= \gamma I(n)-(\mu_{4}+\sigma)R(n), \end{array}\displaystyle \right . $$

where $X(n)=(S(n),E(n),I(n),R(n))$, and

$$\left \{ \textstyle\begin{array}{@{}l} \frac{S(n+1)-S(n)}{\phi(h)}=\Lambda-f(S(n+1),I(n)) -\mu_{1} S(n+1)+\sigma R(n+1),\\ \frac{E(n+1)-E(n)}{\phi(h)}= f(S(n+1),I(n))-(\mu_{2}+\delta )E(n+1),\\ \frac{I(n+1)-I(n)}{\phi(h)}=\delta E(n+1)-(\mu_{3}+\gamma )I(n+1),\\ \frac{R(n+1)-R(n)}{\phi(h)}=\gamma I(n+1)-(\mu_{4}+\sigma)R(n+1), \end{array}\displaystyle \right . $$

with the denominator function $\phi(h)=\frac{e^{\mu_{1}h}-1}{\mu_{1}}$, and $h>0$ is the time-step size. An important open problem is whether the results obtained in this paper for model (2) can also be extended to these models.

## References

[1] Bai Z, Zhou Y (2012). Global dynamics of an SEIRS epidemic model with periodic vaccination and seasonal contact rate. Nonlinear Anal., Real World Appl..

[2] Gao S, Chen L, Teng Z (2007). Impulsive vaccination of an SEIRS model with time delay and varying total population size. Bull. Math. Biol..

[3] Korobeinikov A (2004). Lyapunov functions and global properties for SEIR and SEIS epidemic models. Math. Med. Biol..

[4] Korobeinikov A (2007). Global properties of infectious disease models with nonlinear incidence. Bull. Math. Biol..

[5] Korobeinikov A, Maini P (2005). Non-linear incidence and stability of infectious disease models. Math. Med. Biol..

[6] Kuniya T, Nakata Y (2012). Permanence and extinction for a nonautonomous SEIRS epidemic model. Appl. Math. Comput..

[7] Melesse DY, Gumel AB (2010). Global asymptotic properties of an SEIRS model with multiple infectious stages. J. Math. Anal. Appl..

[8] Nakata Y, Kuniya T (2010). Global dynamics of a class of SEIRS epidemic models in a periodic environment. J. Math. Anal. Appl..

[9] Zhang T, Teng Z (2007). On a nonautonomous SEIRS model in epidemiology. Bull. Math. Biol..

[10] Mickens RE (2000). Application of Nonstandard Finite Difference Scheme.

[11] Mickens RE (2003). Dynamics consistency: a fundamental principle for constructing nonstandard finite difference scheme for differential equation. J. Differ. Equ. Appl..

[12] Mickens RE (2007). Calculation of denominator functions for nonstandard finite difference schemes for differential equations satisfying a positivity condition. Numer. Methods Partial Differ. Equ..

[13] Allen LJS, Driessche P (2008). The basic reproduction number in some discrete-time epidemic models. J. Differ. Equ. Appl..

[14] Allen LJS, Lou Y, Nevai AL (2009). Spatial patterns in a discrete-time SIS patch model. J. Math. Biol..

[15] Brauer F, Feng Z, Castillo-Chavez C (2010). Discrete epidemic models. Math. Biosci. Eng..

[16] Cao H, Zhou Y (2012). The discrete age-structured SEIT model with application to tuberculosis transmission in China. Math. Comput. Model..

[17] Castillo-Chavez C (2001). Discrete-time SIS models with complex dynamics. Nonlinear Anal. TMA.

[18] Chen Q, Teng Z, Wang L (2013). The existence of codimension-two bifurcation in a discrete SIS epidemic model with standard incidence. Nonlinear Dyn..

[19] Enatsu Y, Nakata Y, Muroya Y (2010). Global stability for a class of discrete SIR epidemic models. Math. Biosci. Eng..

[20] Franke J, Yakubu A (2006). Discrete-time SIS epidemic model in a seasonal environment. SIAM J. Appl. Math..

[21] Franke JE, Yakubu AA (2008). Disease-induced mortality in density-dependent discrete-time S-I-S epidemic models. J. Math. Biol..

[22] Franke JE, Yakubu AA (2011). Periodically forced discrete-time SIS epidemic model with disease induced mortality. Math. Biosci. Eng..

[23] Garba SM, Gumel AB, Lubuma JMS (2011). Dynamically-consistent non-standard finite difference method for an epidemic model. Math. Comput. Model..

[24] Hu Z, Teng Z, Jia C, Chen X (2014). Complex dynamical behaviors in a discrete eco-epidemiological model with disease in prey. Adv. Differ. Equ..

[25] Hu Z, Teng Z, Jiang H (2012). Stability analysis in a class of discrete SIRS epidemic models. Nonlinear Anal., Real World Appl..

[26] Ibeas A, de la Sen M, Alonso-Quesada S, Zamani I (2015). Stability analysis and observer design for discrete-time SEIR epidemic models. Adv. Differ. Equ..

[27] Li J, Ma Z, Brauer F (2007). Global analysis of discrete-time SI and SIS epidemic models. Math. Biosci. Eng..

[28] Luo Y, Gao S, Xie D, Dai Y (2015). A discrete plant disease model with roguing and repanting. Adv. Differ. Equ..

[29] Muroya Y, Bellen A, Enatsu Y, Nakata Y (2012). Global stability for a discrete epidemic model for disease with immunity and latency spreading in a heterogeneous host population. Nonlinear Anal., Real World Appl..

[30] Muroya Y, Enatsu Y (2013). A discrete-time analogue preserving the global stability of a continuous SEIS epidemic model. J. Differ. Equ. Appl..

[31] Muroya Y, Nakata Y, Izzo G, Vecchio A (2011). Permanence and global stability of a class of discrete epidemic models. Nonlinear Anal., Real World Appl..

[32] Salceanu PL, Smith HL (2010). Persistence in a discrete-time stage-structured epidemic model. J. Differ. Equ. Appl..

[33] Sekiguchi M (2010). Permanence of a discrete SIRS epidemic model with delays. Appl. Math. Lett..

[34] Wang L, Cui Q, Teng Z (2013). Global dynamics in a class of discrete-time epidemic models with disease courses. Adv. Differ. Equ..

[35] Wang L, Teng Z, Jiang H (2013). Global attractivity of a discrete SIRS epidemic model with standard incidence rate. Math. Methods Appl. Sci..

[36] Yakubu AA (2007). Alee effects in a discrete-time SIS epidemic model with infected newborns. J. Differ. Equ. Appl..

[37] LaSalle JP (1976). The Stability of Dynamical Systems.

[38] Zhao X-Q (2003). Dynamical Systems in Population Biology.

